# Mainzer-Saldino syndrome is a ciliopathy caused by mutations in the *IFT140* gene

**DOI:** 10.1186/2046-2530-1-S1-O28

**Published:** 2012-11-16

**Authors:** I Perrault, S Saunier, S Hanein, E Filhol, A Bizet, F Collins, M Salih, E Silva, V Baudouin, M Oud, N Shannon, M Le Merrer, C Pietrement, P Beales, H Arts, A Munnich, J Kaplan, C Antignac, V Cormier Daire, JM Rozet

**Affiliations:** 1 INSERM U781 & Department of Genetics, Paris Descartes University, France; 2 INSERM, U983, Paris Descartes University, France; 3 Department of Clinical Genetics,Westmead Hospital, Sydney, Australia; 4 Division of Pediatric Neurology, King Khalid University Hospital, Riyadh, Saudi Arabia; 5 Department of Ophthalmology, Coimbra University Hospital, Portugal; 6 Department of Nephrology, CHU Robert Debré, Paris, France; 7 Department of Human Genetics, Radboud University Nijmegen Medical Centre, Nijmegen, the Netherlands; 8 Clinical Genetics Service, City Hospital, Nottingham, UK; 9 Department of Pediatry, American Memorial Hospital, CHU Reims, France; 10 Molecular Medicine Unit, University College London (UCL) Institute of Child Health , UK

## Introduction

Ciliopathies is an emerging class of genetic disorders due to altered cilia assembly, maintenance or function. Syndromic ciliopathies affecting bone development have been classified as skeletal ciliopathies. Mutations in genes encoding components of the intraflagellar transport (IFT) complex A, that drives retrograde ciliary transport, are a major cause of skeletal ciliopathies. Mainzer-Saldino syndrome (MSS) is a rare disorder characterized by phalangeal cone-shaped epiphyses, chronic renal failure and early-onset severe retinal dystrophy.

## Methods and results

We collected 16 families presenting three diagnostic criteria of MSS. Through ciliome re-sequencing combined to Sanger sequencing, we identified *IFT140* mutations in seven MSS families. The effect of the mutations on IFT140 localization was assessed using flagged-IFT140 mutant proteins which showed a partial to nearly complete loss of basal body localization associated with an increase of cytoplasm staining while the wild-type Flagged-IFT140 protein predominantly localized to the basal bodies in RPE1 cells. To assess the impact of *IFT140* mutations on ciliogenesis, abundance and morphology of primary cilia were studied in cultured fibroblasts of patients and detected absent cilia in a high proportion of patient cells compared to controls. Ciliary localization of anterograde IFTs were altered in MSS patient fibroblasts supporting the pivotal role of IFT140 in proper development and function of ciliated cells.

## Conclusion

Here we report on compound heterozygosity or homozygosity for *IFT140* mutations in seven MSS families. After Sensenbrenner and Jeune syndromes, MSS is the ultimate skeletal ciliopathy ascribed to IFT disorganization.

